# Application of individual and integrated pollution indices of trace elements to evaluate the noise barrier impact on the soil environment in Wrocław (Poland)

**DOI:** 10.1007/s11356-022-23563-y

**Published:** 2022-11-12

**Authors:** Anna Hołtra, Dorota Zamorska-Wojdyła

**Affiliations:** Faculty of Environmental Engineering, Wrocław University of Science and Technology, Wybrzeże Wyspiańskiego 27, 50-370 Wrocław, Poland

**Keywords:** Noise barrier, Soil contamination, Trace element, Road traffic, Pollution indices, Cluster analysis

## Abstract

Trace elements emitted by road transport cause long-term contamination of road soils. This study showed the effect of the noise barrier on the spatial distribution of metals in soils at the City Centre Ring Road in Wrocław (Poland). Samples were taken along the barrier every 150 m and across the screen at distances of 0.5, 2.5, 5 (only on the left side), 10, 20, and 50 m from the noise screen on both sides of road no. 5. The contamination of soils was assessed with the use of commonly used individual pollution indices, integrated pollution indices based on the PI index or metal concentrations, and a potential ecological risk index (RI). The geoaccumulation index (Igeo) and the single pollution index (PI) indicate moderate contamination of soils by Cu, Pb, and Zn at most measuring points. The significant risk was reported from Cd in all samples. Integrated pollution indices, especially the PI_Nemerow, PI_avg, and RI confirm the poor quality of studied soils. The presence of elements in soil is related to anthropogenic factors. The spatial distribution of pollution indices and results of statistical analysis (HCA) indicate that Cr and Ni are of natural origin. Remaining elements (Cd, Pb, Cu, Zn) are of mixed origin with a large share from car transport (the significant Spearman’s correlation coefficients). In the case of cadmium, dendograms suggest the existence of an additional anthropogenic source, which is also confirmed by moderately strong correlations of this element with other metals.

## Introduction

The rapid development of road transport is a global environmental problem. The express and safe access to the destination, often via highways, causes increased noise emissions (Can and Aumond 2018; Li et al. 2016; Freitas et al. 2012) and pollutants into the environment (De Silva et al. 2021, 2016; Bernardino et al. 2019; Wang and Zhang 2018; Werkenthin et al. 2014; Yan et al. 2013). To eliminate the noise nuisance, noise screens are installed, which allow for the reduction of sound intensity to values allowed by legal regulations (CEDR 2017). Road barriers can also change the dispersion of pollutant emissions from road transport into the atmosphere (Ghasemian et al. 2017; Venkatram et al. 2016; Amini et al. 2016; Jeong 2015; Hagler et al. 2012, 2011; Baldauf et al. 2008). Aluminum cassette screens are a popular solution in Poland. This type of barrier was installed in the Osobowice district in Wrocław, Poland, next to the national road no. 5, which has been in use for over 10 years, and is in the northern part of the Wrocław City Centre Ring Road. The traffic intensity in both directions of the Jana Nowaka-Jeziorańskiego Street on the Śródmiejska Ring Road is on average 1010 vehicles per hour during the morning and afternoon rush hours (7–8 AM and 4–5 PM). During this time, an average of 3500 vehicles per hour pass through the Millennium Bridge, which is an extension of the above-mentioned street (KBR Report 2018). The screen installed along the Nowaka-Jeziorańskiego Street is designed to reflect sound and absorb acoustic waves due to a special filling. However, this type of noise barrier can cause permanent and irreversible changes in soil quality due to washing away pollutants from the screen surface (Różański et al. 2017; Karbowska et al. 2017; Świetlik et al. 2013). These are mainly dust particles containing, among others, trace elements that occur at low altitude in the living zone of humans, animals, and plants. Due to their small size, they can stay in the atmosphere for a very long time and spread over long distances, which is determined by the speed and direction of the air flow (Charlesworth et al. 2011). Heavy metals from anthropogenic sources tend to be more mobile in the soil than naturally occurring forms of metals in the bedrock (Wuana and Okieimen 2011). The main threats to the environment and health are trace elements, such as cadmium, chromium, copper, nickel, and the main sources are primarily industrial emitters, low emission of solid fuel combustion products from individual sources, and road traffic (Amato et al. 2011; Duong and Lee 2011; Wei and Yang 2010; Zechmeister et al. 2005). The analysis of the impact of road transport on the soil contamination with trace elements showed significant correlations between the content of trace elements in road soils and vehicle speed, road age, and traffic density (De Silva et al. 2016). Higher concentrations of cadmium, copper, lead, zinc, chromium, and nickel are recorded on medium- to older-aged roads (Hołtra and Zamorska-Wojdyła, 2018; De Silva et al. 2016).

Sources of elements are fuel leaks and car exhaust emissions, engine oil and lubricants leaks, car tires and road surface wears, brake pads and discs wears, and exhaust catalyst wear. An important source of zinc is abrasion of the tread of car tires and galvanized vehicle parts. In the case of express roads, the source of zinc is a seasonal maintenance, especially of galvanized metal elements of the technical infrastructure. Car tire wears are a source of manganese. The elements of a frictional brake system (brake blocks) and clutch, corrosion of radiators and chassis, and engine and exhaust system operation are considered to be a source of increased concentration of copper in road soils, and to a lesser extent cadmium, chromium, iron, nickel, lead, and zinc. Chrome car parts are the source of chromium in dust. Leakages of fuel, oils, lubricants, or operating fluids constitute a smaller share of the mentioned metals. Oils and greases are primarily a source of nickel, copper, and cadmium. A source of chromium and nickel is also the abrasion of road surfaces. Despite using of unleaded petrol, an increased level of lead in soils is still observed, which is caused by exhaust gases, and brake lining and car tire wear processes (Charlesworth et al. 2011; Johanssona et al. 2009; Zechmeister et al. 2005). Recently, introduced metals in automotive technology, such as antimony in brake pads, manganese in fuel, or platinum metals (platinum, palladium, rhodium) as an active catalyst layer, are present in roadside dust and soils next to new roads (De Silva et al. 2016). The contamination of roadside soils also results from the transport of inadequately protected loose materials, corrosion of road infrastructure, and the use of sand with road salt to keep roads clean in winter (Aljazzar and Kocher 2016; Werkenthin et al. 2014).

The purpose of this study was to assess the level of contamination in the soil surface layer with metals (i.e., zinc, copper, lead, chromium, nickel, and cadmium) along the noise screen, and to determine the impact of the barrier on the spatial distribution of soil pollutants up to 50 m on both sides of the screen. Moreover, the validity of multi-indices of pollution approach to the soil’s quality was assessed. Individual pollution indices of PI and Igeo; integrated pollution indices calculated from the PI index for metals; and CSI, MERMQ, and RI indices were used. By using a reference geochemical background, comparing similar objects becomes much easier. To our knowledge, the quantitative data for pollution indices of soils with trace elements near noise barriers have not been collected and compared so far. The comprehensive analysis to assess the impact of the technical structure on the dispersion and transport of emissions generated by road traffic to the soil environment has not been performed either. This approach can be considered a universal tool for assessing soil quality and its utility value.

## Materials and methods

### Description of the study area and soil sampling

The noise screen is located along of the section road no. 5 (the Jana Nowaka-Jeziorańskiego Street in Wrocław city, West Poland) and is a part of the Wrocław City Centre Bypass. A steel barrier, approximately 4 m high and 1.7 km long, is located on the west side of the road no. 5, between the Millenium Bridge and the railway viaduct. The GPS coordinates of the location of the road segment with the screen are from 51°08′13.9″N 17°00′03.1″E to 51°09′08.0″N 17°00′15.9″E.

The studied section of the road no. 5 runs north from the exit at the Millennium Bridge over the Odra River up to the curve where the Jana Nowaka-Jeziorańskiego Street turns to the east and crosses road no. 342. Road no. 5 is a four-lane road, with a green belt between communication routes leading in the opposite direction and the road surface is asphalt. There is a paved shoulder in the road strip, separated by a curb and runoff of surface waters. Measurement of traffic on the Millennium Bridge in 2018 indicated 45,220 vehicles per day (KBR Report 2018). Due to the lack of exits from the main road, it can be assumed that the traffic intensity is uniform.

The Wrocław-Różanka district is a new district of the city, transformed from a rural town. The road no. 5 with the noise screen has been in use since 2010. Currently, the Różanka district has apartment blocks, schools, offices, and institutions heated from the municipal heating plant. The remains of rural buildings are villas and single-family houses fired with solid fuels. There are no large shopping centers or an industrial zone on the site. In the immediate vicinity of the tested section of the road no. 5, there are allotment gardens, a railway line and embankments, and slopes covered with vegetation.

Soils in this area are classified as alluvial soils and soil-glial soils, which are under the constant influence of the high groundwater level. They are usually characterized by a compact grain size of medium and heavy clays, most often silty clays, and clay dust. Sometimes the dust-clay layer is so small that a sandy base is found at the bottom (Kabała and Chodak 2002).

In Wrocław, winds from the western direction dominate, which is on an average of 51% a year. From September to January, the frequency of south-west winds increases. Winds from eastward represent an average of 32% a year and mostly occur in April. Winds from the north-east and north rarely appear (approx. 7% a year) (Woś 2010).

**Fig. 1:**
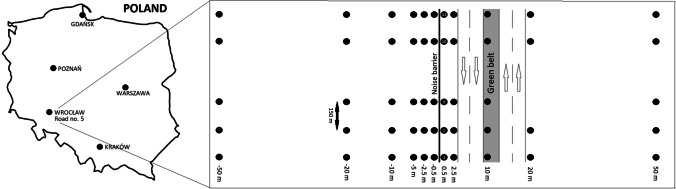
Location of the sampling points

Along the noise screen, 11 points were selected at intervals of ca. 150 m (Fig. 1). The collection of samples was also carried out in the direction perpendicular to the screen. On the left side of the noise barrier (the west side), 6 sampling sites were set, respectively: − 0.5 m, − 2.5 m, − 5 m, − 10 m, − 20 m, and − 50 m from the screen. On the right side of the screen (the east side with four lanes of Road no. 5), 5 sampling sites were set, respectively: 0.5 m, 2.5 m, 10 m, 20 m, and 50 m from the screen. A total of 121 soil samples were collected.

The surface soil samples were taken from a depth of up to 20 cm in accordance with guidelines of the Minister’s Regulation of September 1, 2016, on the method of assessing soil contamination (Regulation 2016). At each location, 6 soil subsamples were collected in an area of 2–3 m^2^ and then mixed into one representative sample for each sampling point. The soil material was collected in July 2019.

### Laboratory and statistical analysis

The air-dried soil samples were ground in a mortar and sieved through mesh of 1 mm. Measurements of the electrical conductivity and pH of the solution were carried out in the following system: the soil mass to suspension volume in a stoichiometric ratio of 1:2.5 (m:v) according to PN–ISO 10390 (1997) using a multifunction meter CX-701 made by Elmetron. The pH measurements were made in a 1 mol/dm^3^ of potassium chloride solution (pH-KCl). Soils for spectroscopic analysis were weighed in an amount of about 0.2 g on an AUW 120D Shimadzu analytical balance. The soil samples were flooded with 8 ml of 65% nitric acid (V) Suprapur of Merck. The digestion process was carried out in a closed microwave etching system using a Start D Milestone device. The process of decomposition of organic substances in soil samples was carried out under the conditions of a linear temperature increase to 220 °C with the use of 800 W microwave power. The total content of copper, zinc, lead, chromium, nickel, and cadmium was determined in filtrates after mineralization. To determine concentrations of trace elements, the flame and electrothermic atomic absorption spectrometry methods using a Thermo Solaar iCE 3500 device according to the PN–ISO 11047 (2001) method was used. Certified reference materials of analyzed chemical elements (CRM) from Sigma Aldrich were applied to the elemental analysis. The reagent blank samples were used to check the instrument readings. The limits of detection were estimated based on three times the standard deviation for digestion blanks. The accuracy of the determination was controlled using the method of the standard addition. The percentage of the recovery was 94–98%. The AAS results were verified through the standard deviation, the coefficient of variation, and the confidence interval. The accepted results of the coefficient of variation for a given point were within 10%. Statistical analysis was made based on the Student *t*-test with the number of degrees of the freedom equaling 5 and the *p*-value being 0.05. Chemical analysis was been carried out in the certified Laboratory of Environmental Research in the Faculty of Environmental Engineering at the Wrocław University of Science and Technology.

The content of trace elements in the soil was calculated as the average value with the standard deviation for six independent environmental samples from one sampling site. The hierarchical cluster analysis (HCA) was performed with Ward’s algorithm and the squared Euclidian distance. The average values of the pollution index for a given location were used. The statistical analysis was carried out using the program Statistica and Microsoft Excel. The data was checked for normal distribution of the Shapiro–Wilk’s *W*-test. Tests of significance were made at 95% confidence level. The Mann–Whitney *U*-test was used for the data which did not show the normal distribution. The Spearman’s rank correlation coefficients were calculated for elemental concentrations for all samples. Correlations were considered strong when higher than 0.7, and the *p*-value was 0.05. Pearson’s correlation coefficient (*p* < 0.05) was used to explore relationships between the total content of analyzed trace elements and soil parameters. The presented research methodology and statistical analysis were used in our earlier works (Hołtra and Zamorska-Wojdyła 2018, 2020).

### Environmental pollution indices

Based on the content of trace elements in soils, pollution indices were calculated to show the degree of environmental pollution with heavy metals and related threats. The calculation of pollution indices was conducted using the elemental composition from the upper continental crust (UCC) recommended by Rudnick and Gao (2003) in mg/kg: 28 (Cu), 67 (Zn), 17 (Pb), 47 (Ni), 92 (Cr), and 0.09 (Cd). The values of this reference geochemical background represent lithogenic contents of trace elements without the influence of pedogenic processes. Calculated pollution indices provide a more universal character by using proposed reference UCC values.

The following pollution indices were discussed by Kowalska et al. (2018). The geoaccumulation index is calculated using the following formula: $\mathrm{Igeo}={\mathrm{log}}_{2}(\mathrm{Cn}/1.5\cdot \mathrm{Bn})$, where Cn denotes the concentration of the metal, and Bn denotes the value of the background. Seven of the Igeo classes (from 0 to 6, respectively) are distinguished: unpolluted (Igeo < 0), unpolluted to moderately polluted (Igeo = 0–1), moderately polluted (Igeo = 1–2), moderately to highly polluted (Igeo = 2–3), highly polluted (Igeo = 3–4), highly to extremely polluted (Igeo = 4–5), and extremely high polluted (Igeo > 5).

The single pollution index (PI) for an individual trace element was defined by the following formula: $\mathrm{PI}=\mathrm{Cn}/\mathrm{Bn}$. Five PI classes are distinguished: absent (PI < 1), low pollution (PI = 1–2), moderate pollution (PI = 2–3), strong pollution (PI = 3–5), and very strong pollution (PI > 5).

The values of individual pollution indices for each trace elements were used for the calculation of the integrated pollution. The average pollution index (PI_avg) can be defined as follows:$\mathrm{PI}\_\mathrm{avg}=\mathrm{PI}\_\mathrm{sum}/n$, where *n* denotes the number of studied trace elements (*n* = 6). The sum of contamination (PI_sum) of six trace elements is defined with the following formula: $\mathrm{PI}\_\mathrm{sum}=\mathrm{\Sigma PI}$. The vector modulus of pollution index (PI_vector) is defined as $\mathrm{PI}\_\mathrm{vector}=\sqrt{{1/n\cdot \mathrm{\Sigma PI}}^{2}}$.

The pollution load index (PLI) is a geometric average of the PI index, based on the following formula: $\mathrm{PLI}=\sqrt[n]{\mathrm{PI}1\cdot \mathrm{ PI}2\cdot \mathrm{ PI}3\cdot \dots \cdot \mathrm{PIn}}$. Three classes of pollution are distinguished, respectively: perfect quality (PLI < 1), baseline levels of pollution (PLI = 1), and deterioration of quality (PLI > 1).

The Nemerow pollution index (PI_Nemerow) is calculated by the following formula: $\mathrm{PI}\_\mathrm{Nemerow}=\sqrt{({\mathrm{PI}\_\mathrm{avg}}^{2}+{\mathrm{PI}\_\mathrm{max}}^{2})/2}$. The following quality of soil is distinguished (five of PI_Nemerow classes): clean (< 0.7), warning limit (0.7–1), slight pollution (1–2), moderate pollution (2–3), and heavy pollution (> 3).

The background enrichment factor (PIN) is defined as $\mathrm{PIN}=\Sigma ({{\mathrm{PI}}_{\mathrm{class}}}^{2}\cdot \mathrm{Cn}/\mathrm{Bn})$. Five classes of contamination are distinguished, respectively: clean (0–7), trace contaminated (7–95.1), lightly contaminated (95.1–518.1), contaminated (518.1–2548.5), and highly contaminated (≥ 2548.5).

The potential ecological risk (RI) is defined as the sum of ecological risk factors (Er) for all metals in each sample, according to the following formula: $\mathrm{RI}=\mathrm{\Sigma Er}$. Four categories of this index are distinguished: low (RI < 90), moderate (RI = 90–180), strong (RI = 180–360), very strong (RI = 360–720), and highly strong (RI > 720). The ecological risk factor for a single trace element can be calculated using modified Hakanson’s (1980) equation: $\mathrm{Er}=\mathrm{Tr}\cdot \mathrm{PI}$, according to Kowalska et al. (2018). The Tr is the toxic-response factor for a given metal: Cu = 5, Zn = 1, Pb = 5, Ni = 5, Cr = 2, and Cd = 30 (Hakanson 1980). The following categories of pollution are distinguished: low (Er < 40), moderate (Er = 40–80), considerable (Er = 80–160), high (Er = 160–320), and very high (Er > 320) after Zgłobicki et al. (2018).

The contamination security index (CSI) is calculated based on the formula:${\mathrm{CSI}=\Sigma W((\mathrm{Cn}/\mathrm{ERL}}^{1/2}){+(\mathrm{Cn}/\mathrm{ERM}}^{2}))$, where *W* is the computed weight of each trace element: 0.075 (Cu, Zn), 251 (Pb), 215 (Ni), 134 (Cr), and 0.25 (Cd). Values of the effect range low (ERL) and the effects range median (ERM) are, respectively, 34/270 (Cu), 150/410 (Zn), 46.7/218 (Pb), 20.9/51.6 (Ni), 81/370 (Cr), and 1.2./9.6 (Cd). An interpretation of values is as followed (nine of CSI_classes): uncontaminated (CSI < 0.5), very low contamination severity (CS I = 0.5–1), low contamination severity (CSI = 1–1.5), low-to-moderate contamination severity (CSI = 1.5–2), moderate contamination severity (CSI = 2–2.5), moderate-to-high contamination severity (CSI = 2.5–3), high contamination severity (CSI = 3–4), very high contamination severity (CSI = 4–5), and ultra-high contamination severity (CSI > 5).

The probability of toxicity (MERMQ) is calculated according to the formula:$\mathrm{MERMQ}=(\mathrm{\Sigma Cn}/\mathrm{ERM})/n$. An interpretation of this index values is as follows: low risk level (9%) probability of toxicity (MERMQ < 0.1), medium risk level (21%) probability of toxicity (MERMQ = 0.1–0.5), high risk level (49%) probability of toxicity (MERMQ = 0.5–1.5), and very high-risk level (76%) probability of toxicity (MERMQ > 1.5).

## Results and discussion

The standard distribution of traffic pollution in soils along roads (without noise barriers) decreases with distance and reaches the natural level at 30–50 m from the edge of the road (Werkenthin et al. 2014; Massas et al. 2013; Kluge and Wessolek 2012). Zechmeister et al. (2005) suggest a much larger range road pollution dispersion by air, even up to 250 m from the edge of the road. Many authors confirm soil contamination with heavy metals in the road lane, including based on the value of the most frequently chosen of the Igeo index for assessing the quality of road soils (Hołtra and Zamorska-Wojdyła 2018; Trujillo-Gonzalez et al. 2016; Massas et al. 2013).

Rybak et al. (2021) and Hołtra and Zamorska (2018) confirmed that road transport is the main source of metals in tested road soils in Wrocław. Świetlik et al. (2013) additionally emphasized the importance of low emissions from individual furnaces (solid fuel combustion), which deteriorates the quality of air and urban soils. Particularly high levels of dust in the air and high concentrations of toxic metals in the air (metals associated with PM) are recorded in densely populated cities, and Poland is considered the infamous leader in Eastern Europe (Sówka et al. 2019, 2015; Guerreiro et al. 2018; González Ortiz et al. 2017).

The problem of heavy metal input in road soils from road transport at the metal noise barrier and its further vicinity has not been widely analyzed so far (Wawer et al. 2017, 2015; Różański et al. 2017; Hajok et al. 2017). There are well-known and advanced model studies of the air quality concerning the influence of noise barriers on dispersion and transport of pollutants emitted by road traffic. However, there are no studies on deposition of solid pollutants from road emissions on the roadside soil near noise barriers. Baldauf et al. (2008) showed that barriers inhibit lateral air movement, and thus, can reduce wind transmission of pollutants and increase their deposition near the road (in front of the barrier). In addition, noise barriers increase the turbulence of the airflow at the barrier and increase vertical mixing due to the upward reversal of the airflow which, on the windward side, creates a recirculation cavity extending from 3 to 12 m in height of the barrier. Blocking the scattering of chemical elements by noise barriers reduces the concentration of solid particles behind the barrier by up to 50%. Bowker et al. (2007) proved that at greater distances behind the noise screen, higher concentrations of pollutants occur because of vertical rise of the air flowing through the barrier along with a smudge of pollutants. Baldauf et al. (2008) also noted the importance of the wind direction in the transmission of pollutants. They showed that in the case of winds directed along the road, dust concentrations decreased by 15–50% behind the noise barrier.

### Trace elements content in soils

The median levels of selected metals in soils can be arranged in the following order: Zn > Cu > Pb > Cr > Ni > Cd. In the case of maximum levels, Pb and Ni changed the order in the series on the right side of the noise barrier (the east side) (Table 1).

**Table 1:** Descriptive statistics of metals in soils [mg/kg]

Parameter	Zn	Cu	Pb	Cr	Ni	Cd	Noise barrier	Zn	Cu	Pb	Cr	Ni	Cd
Min	36.29	42.57	2.22	16.36	20.85	3.15		17.32	36.08	2.34	2.31	0.03	0.19
Max	1034.39	220.04	198.88	137.62	73.69	24.14		1371.15	318.26	361.83	78.06	88.21	7.79
Mean	241.25	86.00	58.64	56.08	45.05	6.51		302.16	91.83	53.73	34.77	27.85	2.11
SD	200.83	36.31	50.55	26.23	11.98	2.71		254.91	48.85	58.77	17.34	18.90	1.68
Q1	111.22	63.16	26.55	39.53	37.67	5.39		171.70	65.19	12.47	22.31	11.06	0.98
Med	166.71	75.23	39.63	51.29	44.39	5.96		221.53	81.72	34.88	32.62	25.54	1.60
Q3	278.25	93.25	83.52	72.22	53.59	6.71		335.10	99.46	79.67	45.39	40.47	2.61
Skewness	2.06	1.87	1.39	0.94	0.36	4.46		2.35	2.64	2.83	0.34	0.74	1.83
Kurtosis	4.45	3.74	1.29	0.89	− 0.21	26.76		6.34	8.83	12.47	− 0.25	0.53	3.39
CV	0.83	0.42	0.86	0.47	0.27	0.42		0.84	0.53	1.09	0.50	0.68	0.80
*p*-value	< 0.05	< 0.05	< 0.05	< 0.05	> 0.05	< 0.05		< 0.05	< 0.05	< 0.05	> 0.05	< 0.05	< 0.05
*n*	66	66	66	66	66	66		55	55	55	55	55	55

**Fig. 2:**
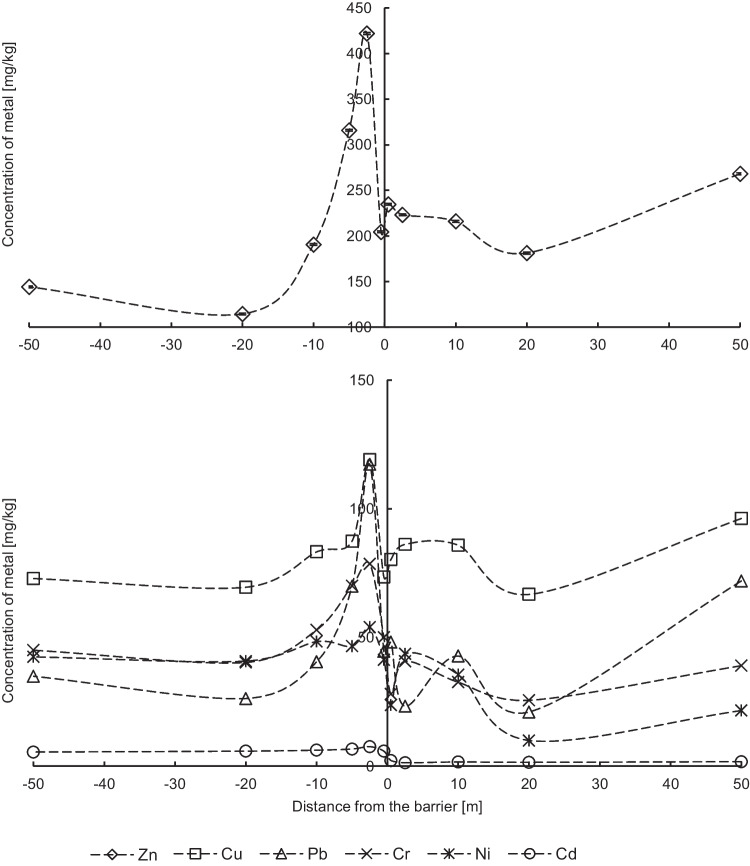
Metal content in soils (median values) along with the distance from the noise barrier (0 m)

On the left side of the screen (the west side), average concentrations of chromium and nickel are 1.7 times higher than on the right side of the barrier (the east side), and the concentration of cadmium is about 3.8 times higher. In contrast, average levels of zinc, copper, and lead are similar on both sides of the screen. On the left side of the noise barrier, at the location of 2.5 m from the screen, the highest values of median and maximum for tested metals in soils were recorded, except for the maximum concentration of Cd, which was twice at 10 m from the screen (Fig. 2). On the right side of the screen, the highest values of median and maximum were recorded at 50 m from the screen, except for Ni which was twice as high at 2.5 m.

On the left side of the noise screen, the maximum value for a given metal was higher than the median value by about 2.5 times for Zn, 1.9 times for Cu, 1.7 times for Pb and Cr, 1.4 times for Ni, and 1.6 times for Cd. On the right side of the screen, the difference between the maximum value and the median was higher and it was 5.1 times for Zn, 3.3 times for Cu, 5.0 times for Pb, 2 times for Cr and Ni, and 3.6 times for Cd, respectively.

Differences in the spatial distribution of metals may result from the wind and the dominance of winds blowing from the left (the west side of the barrier) to the east, and the presence of the barrier itself, which blocks the horizontal air flow in the field. Disturbances in the distribution of concentrations of elements in the soil due to the presence of the noise barrier are best seen on the example of zinc and copper (i.e., metals with the highest concentrations in the soil) (Fig. 2). On the right side of the screen (in the road lane), an increased level of metals in soils is observed, especially at 0.5 m to 10 m, which confirms that the barrier partially blocks the flow of emitted road pollutants in the communication route. Thus, the dispersion of pollutants on the east side of the screen is mainly due to road and car traffic. In front of the barrier (on the left side), a great increase of metal concentrations is observed right next to the screen, which may be caused by particle falling after air masses collide with the noise barrier as the windward side for dominant westerly winds (70%). In the case of less frequently winds blowing from the east (30%), such a large impact of the barrier on the retention of road dust in the traffic route line (in the close vicinity of the screen) is not observed. Increased concentration of metals in the vicinity of the noise screen may also be due to the material used for the construction of the road and the roadside, as the soil near the road is of unknown origin with difficult to determine degree of trace element contamination. At more than 20 m from the screen, an increase in Zn, Cu, and Pb concentrations is observed on both sides, which proves that road dust particles containing trace elements are transported there by the wind.

Sanders et al. (2003) estimated that about 30% of road dust falls on the road. Thus, not a total input of metals from road traffic loads the soil environment bordering the road. Concentrations of Zn (12 times), Cu (2 times), and Cr (3.5 times) in our soils at the noise barrier were much lower than in road dust collected from the surface of the screen by Świetlik et al. (2013). In our research, higher concentrations of Ni (1.2 times) and Pb (1.4 times) were noted.

Karbowska et al. (2016) also examined contents of cadmium and lead in dust retained on the surface of noise barriers located along the S5 expressway near Poznań (Poland). They noticed a positive correlation between the barrier length and the total concentration of tested metals on the barrier. Previous reports show that not only the structure of the barrier, its length, and height but also weather conditions (e.g., the direction and speed of the wind) significantly affect the dispersion of pollutants near the road. In addition, rainfall causes the dust accumulated on noise barriers to be washed away into the surrounding soil.

Hajok et al. (2017) confirmed that the noise barrier limits the movement of heavy metals from linear emission and has a statistically significant effect on the distribution and content of metals in the soil. The distribution of metals in soils in the city of Tychy (Upper Silesia, Poland), on both sides of the S86 expressway, resembles the case from Wrocław analyzed in this paper. The highest concentrations of metals by Hajok et al. (2017) were observed at 1 m from the screen and were, respectively, Pb 47 times, Zn 23 times, and Cd 3.0 times higher than in Wrocław. At 1 m from the edge of the road without the screen, metal levels were, respectively, Pb 5 times, Zn 3 times, and Cd 1.5 times higher than in Wrocław at the same sampling distance. The highest concentrations of metals in Tychy on the side of the road without a screen were recorded at 8 m from the edge of the road for Cd (2.25 mg/kg), Pb (450 mg/kg), and Zn (1000 mg/kg).

Wawer et al. (2017) conducted research on spread and deposition of traffic pollution around the Upper Silesian conurbation (Upper Silesia, Poland) related to the impact of noise barriers along the Central Katowice-Gliwice Highway between Chorzów and Zabrze cities. The accumulation of metals (Zn, Cu, Pb, Cr, Ni, and Cd) in the road lane was noted, with a special accumulation of elements in the soil near the edge of the road, which results from intensive road traffic and water runoff from the road surface. A decrease in the level of metals at the barrier itself (0.15 cm) can also be observed. On the other side of the screen (0.15, 10, and 15 m), concentrations of trace elements were much lower and remained at a similar level at studied distances. Behind the screen, the increased concentration of pollutants in soils was recorded at the maximum tested distance (15 m) from the road barrier. This confirms that noise barriers disturb the spatial distribution of traffic pollution because no decrease in metal concentrations is observed with increasing distance from the edge of the road. In our research in Wrocław, higher levels of Cu (2 times), Cr (5 times), Ni (4 times), Cd (3 times), and lower contents of Zn and Pb (5 times) were recorded at distances of 10–20 m, respectively, on the left side of the screen. In the scope of the research presented by Wawer et al. (2017), it is difficult to show similarity with our spatial distribution of metals at greater distances from the noise barrier. The attention is drawn to the presence of apartment blocks at 15 m from the road in Upper Silesia, which are heated by a collective district heating system. The proximity of apartment blocks along the traffic route may cause no pollutants and their accumulation on the road lane. In the paper by Wawer et al. (2017), there is no information on the prevailing wind direction in the study area, which is important for the transmission of road pollutants, especially in the case of winds directed along the road with high buildings, as indicated by Baldauf et al. (2008). The contribution of metals in soils at various barriers was analyzed for steel panels with galvanized structural elements, noise screens such as green walls, Plexiglass, earth, and sawdust concrete embankments (Wawer et al. 2017). The authors showed that steel panels pose an additional hazard to the environment in the winter season due to corrosion in contact with road salt, which leads to an increase in the zinc content.

Różański et al. (2017) paid attention to the disturbance of the spatial distribution of trace elements in soils near the screen installed outside the built-up area along the A1 Grudziądz motorway (Poland). Placing the screen caused the accumulation of Zn, Pb, and Ni just behind the road barrier, in the closest tested distance from the barrier (5 m). Comparison with our results for the same distance from the barrier shows much higher concentrations of trace elements in soils in Wrocław, respectively, for Zn at 9 times, Pb at 43 times, Ni at 5 times, Cu at 18 times, Cr at 3 times, and Cd at 77 times. It turns out that in Różański et al. (2017), the concentration of Cd closest to the screen (5 m) was the lowest among all tested distances. At distances of 10, 25, and 50 m from the barrier, the concentration of cadmium in arable soils was 15 to 25 times lower than in our research. Much higher concentrations of metals in soils in Wrocław may result from a twice as longer use of road no. 5 than the A1 motorway in Grudziądz (Poland). In our research, we also observe an increase in metal concentrations at the screen and at 50 m from the screen, with differences on both sides of the barrier (Fig. 2). The content of Zn, Cu, and Pb at 50 m on the right side of the screen (the east side) in Wrocław is twice as high for Zn and Pb and 1.5 times higher for Cu than at 50 m on the left side of the screen (the west side). In the case of Cr, Ni, and Cd, higher concentrations of metals were recorded on the left side of the screen at tested distances (50 m), respectively, 1.5, 2, and 3 times greater than on the right side of the barrier. Różański et al. (2017) explained the accumulation of metals at a screen by turbulent air movement with suspended particles of dust and aerosols with adsorbed trace elements. They also state that only coarse dust fractions can fall by gravity in the distance of up to 10 m from the barrier. Above 10 m from the screen, the speed of air masses gradually decreases, and at 50 m from the screen, it is so low that even fine dust particles can settle on the surface of the soil. Różański et al. (2017) do not mention the direction of the winds in the study area, which may affect the spatial distribution of metals in soils.

According to Polish guidelines, the study area along the public road in Wrocław can be classified as not contaminated with analyzed metals due to the lack of exceedances of allowable metal values for soil of group IV (Regulation of the Polish Ministry of Environment 2016). In the area of wastelands, which are in some places wooded and shrubby (soils of group III according to the Polish regulation), the maximum value for zinc was exceeded by about 1.4 times at 50 m on the right side (the east side) of the noise screen.

### Correlation analysis

Based on obtained results, a strong spatial variability of trace metal content in soils in the studied area was found (Table 1). The highest values of CV were recorded for Pb, Zn, and also Cd on the right side of the noise barrier (> 80%), which means a high level of spatial heterogeneity (strong anthropogenic influence). The coefficients of variation for Cr, Cu, and Ni amounted to about 50% and below. The lack of spatial homogeneity is also confirmed by the *p*-value of the Shapiro–Wilk’s *W*-test. Only two elements can pass the normal distribution: Ni (*p*-value = 0.408) on the left side (the west side) and Cr (*p*-value = 0.508) on the right side (the east side) of the screen (Table 1).

When analyzing results of metal concentrations along the screen (along the road line), the average variability of Cr, Cd, Cu, and Zn concentrations (CV < 45%) was found on the left side (the west side) of the screen in the studied soil population for assumed distances from the barrier. A low variability of concentrations was obtained for Ni. Similar concentrations of the metal in soils collected along the road at the same distance confirm one common source of the element’s emission. On the right side of the screen, there was a strong variability of metal concentrations (high concentration heterogeneity) at all tested distances from the screen (CV > 60%). This is due to the accumulation of road pollutants in the road lane, which are not blown out by the wind moving over the noise barrier.

The frequency histograms for five studied metals in soils confirm the deviation from the Gaussian curve (Table 1). Positive kurtosis indicates a concentration of scores around the mean value and the presence of a small number of outliers. Positive skewness values suggest that the arithmetic mean values are higher than median values. The exceptions are nickel and chromium with negative kurtosis, with small absolute values for this parameter, which is confirmed by a distribution close to the normal for both elements.

In studied soils, there were significant correlations between the total metal content (*p* < 0.05 of Mann–Whitney *U*-test) except for the Pb–Cr and Pb–Ni pairs (Table 2). Strong (0.7–0.9) or moderate (0.4–0.7) Spearman’s correlation was identified between most metals, which may suggest that these metals have a common source of origin related to road traffic (exhaust emissions, brake and tire wear, road abrasion, corrosion of road structures, and car parts).

**Table 2:** Spearman’s correlation analysis (*p* < 0.05; *p > 0.05)

	Zn	Cu	Pb	Cr	Ni	Cd	Noise barrier	Zn	Cu	Pb	Cr	Ni	Cd
Zn	-	0.88	0.77	0.77	0.68	0.69		-	0.88	0.75	0.80	0.72	0.71
Cu	-	-	0.70	0.90	0.82	0.72		-	-	0.70	0.79	0.74	0.64
Pb	-	-	-	0.57*	0.45*	0.54		-	-	-	0.70*	0.54	0.69
Cr	-	-	-	-	0.79	0.62		-	-	-	-	0.69	0.41
Ni	-	-	-	-	-	0.67		-	-	-	-	-	0.44

The research done by Wawer et al. (2017) pointed to specific structural elements of vehicles as a source of metals from road emissions to the atmosphere and soils. Strong and very strong Pearson’s correlation values between metal pairs were predominate, except for Cd with Cu, Cr and Ni, and Zn with Cu and Cr (< 0.5, a moderate or weak correlation). The authors explain the strong correlations of Pb with Cu, and Ni and Cd due to the wear of brake linings and tires. Additionally, strong relationships of Fe, Mn (> 0.7) with other elements, and strong correlations of the magnetic susceptibility of solid contaminants with metals confirm their origin from the wear of brake system components.

Świetlik et al. (2013) received a significant Pearson’s correlation value only for two pairs, Cu–Cr (0.55) and Zn–Cr (0.43), in road dust collected from the noise barrier by the E77 road in typically agricultural areas devoid of industrial and heating sources of trace metals. This fact has been linked to the wear of brakes, brake discs, drums, and brake linings, which is additionally confirmed by Cu–Mn and Cu–Fe correlations.

### Pollution assessment

#### Individual pollution indices

The median values of individual indices (PI and Igeo) are ranked as follows: Cd > Cu > Zn > Pb > Ni > Cr on the left side (the west side) of the noise screen. In the case of the right side of the barrier (the east side), Cu and Zn changed the order in the series. The maximum values for PI and Igeo indices form a series: Cd > Zn > Pb > Cu > Ni > Cr. The assessment of the degree of soil pollution based on median values, considering geochemical values (the UCC reference background), indicates a very strong pollution of Cd: the 4th or the 6th of Igeo_classes (very strong to extremely high polluted soils) and the 5th of PI_class (very strong pollution) (Table 3). The geochemical background was exceeded for Cd over 66 times on the left side and 17 times on the right side of the barrier, and a very high ecological risk (Er > 320) is observed in 90% of soil samples due to the cadmium contamination. The remaining 10% of samples indicate considerable or high potential ecological risk. Studied trace elements do not contaminate examined soils, which is confirmed by the values of median of Igeo < 1, except for Zn (moderately polluted) on the right side of the noise barrier (the east side). The median values of PI index indicate that soils are moderately polluted by Cu, Pb, and Zn (strong pollution). The differences between the Igeo and PI classes result from the different ways of calculating these indices, because in the case of Igeo index, the conversion factor of 1.5 and the logarithmic operation to eliminate background variability are used.

**Table 3:** Descriptive statistics of individual pollution indices

Index	Parameter	Zn	Cu	Pb	Cr	Ni	Cd	Noise barrier	Zn	Cu	Pb	Cr	Ni	Cd
PI	Min	0.54	1.52	0.13	0.18	0.44	35.00		0.26	1.29	0.14	0.03	0.01	2.08
	Max	15.44	7.86	11.70	1.50	1.57	268.24		20.46	11.37	21.28	0.85	1.88	86.54
	Mean	3.60	3.07	3.45	0.61	0.96	72.33		4.51	3.28	3.16	0.38	0.59	23.42
	SD	3.00	1.30	2.97	0.29	0.25	30.14		3.80	1.74	3.46	0.19	0.40	18.66
	Q1	1.66	2.26	1.56	0.43	0.80	59.87		2.56	2.33	0.73	0.24	0.24	10.87
	Med	2.49	2.69	2.33	0.56	0.94	66.19		3.31	2.92	2.05	0.35	0.54	17.74
	Q3	4.15	3.33	4.91	0.78	1.14	74.51		5.00	3.55	4.69	0.49	0.86	29.04
	Skewness	2.06	1.87	1.39	0.94	0.36	4.46		2.35	2.64	2.83	0.34	0.74	1.83
	Kurtosis	4.45	3.74	1.29	0.89	− 0.21	26.76		6.34	8.83	12.47	− 0.25	0.56	3.39
	*n*	66	66	66	66	66	66		55	55	55	55	55	55
Igeo	Min	− 1.47	0.02	− 3.52	− 4.60	− 2.56	4.54		− 2.54	− 0.22	− 3.45	− 5.90	− 11.40	0.47
	Max	3.36	2.39	2.96	0.01	0.06	6.69		3.77	2.92	3.83	-0.82	0.32	5.85
	Mean	0.88	0.93	0.63	− 1.50	− 0.71	5.50		1.18	0.99	0.28	− 2.24	− 1.91	3.55
	SD	1.02	0.51	1.40	0.78	0.44	0.38		1.14	0.60	1.62	0.97	1.77	1.13
	Q1	0.15	0.59	0.06	− 1.80	− 0.90	5.32		0.77	0.63	− 1.04	− 2.63	− 2.67	2.84
	Med	0.73	0.84	0.64	− 1.44	− 0.67	5.46		1.14	0.96	0.45	− 2.08	− 1.47	3.52
	Q3	1.47	1.15	1.71	− 0.95	− 0.40	5.63		1.74	1.24	1.64	− 1.60	− 0.80	4.26
	Skewness	0.35	0.86	− 0.52	− 0.98	− 1.12	0.61		− 0.59	0.81	− 0.20	− 1.49	− 2.96	− 0.32
	Kurtosis	− 0.08	0.69	0.11	2.64	3.50	1.98		1.92	1.60	− 0.82	3.09	13.83	0.44
	*n*	66	66	66	66	66	66		55	55	55	55	55	55

**Fig. 3:**
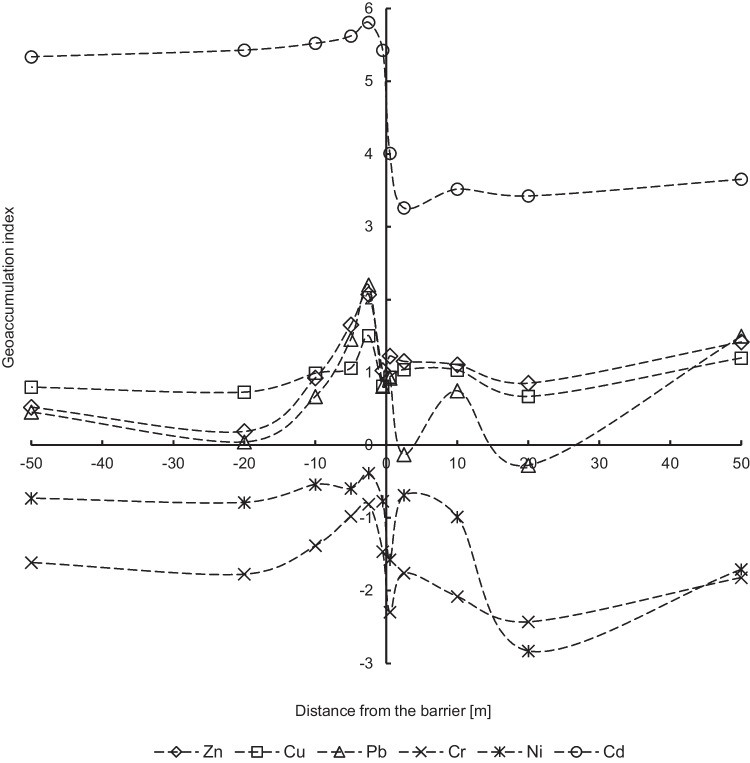
Geoaccumulation index (median values) of metals in soils

**Fig. 4:**
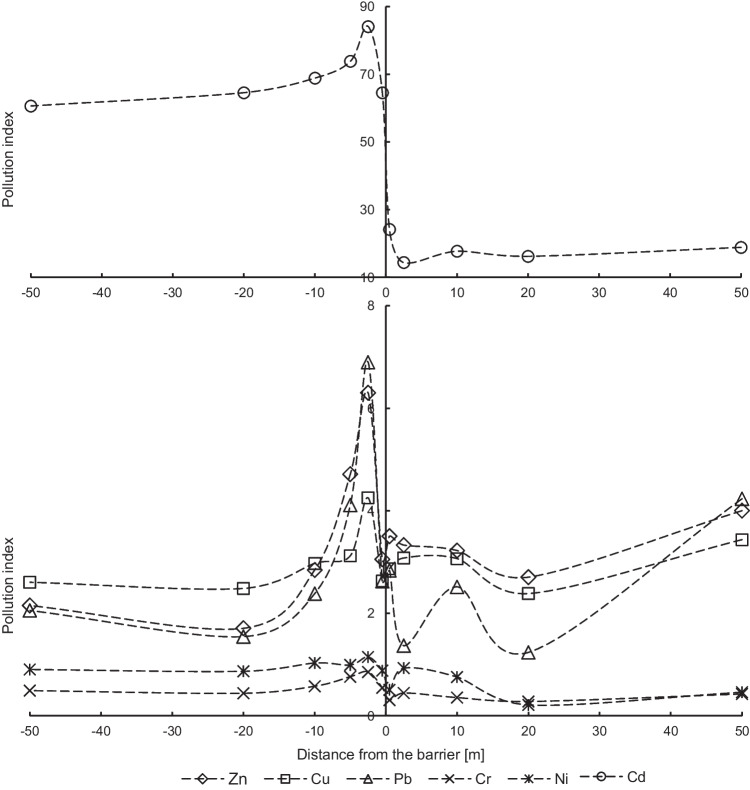
Single-pollution index (median values) of soils with trace elements

The analysis of individual pollution indices with the distance from the noise screen confirms the heterogeneous distribution of metals in the studied area (Figs. 3 and 4). The highest values of median and maximum of individual indices are exactly in the same points for Zn, Cu, and Pb (2.5 m on the left and 50 m on the right from the noise barrier), as values of median and maximum of concentrations of these metals. (Fig. 2). In the case of cadmium, the maximum of Igeo values are similar and are above 5 (the 6th Igeo_class) for each of the tested locations. The median of Igeo values for cadmium is higher on the left side (the west side) of the noise barrier. This is the difference between the two Igeo classes compared to the right side (the east side).

In the remaining measurement points on both sides of the noise screen, the values of individual indices for individual metals are almost at the same level, respectively, the PI values for Zn, Cu, and Pb are in the range from 2 to 3, and the Igeo values are in the range from 0 to 2. On the other hand, the PI values for Cr and Ni are below 1 unit, and for the Igeo parameter there are below 0. Based on both individual indices, except the maximum points, moderate soil pollution of Zn, Cu, and Pb and low soil pollution of Cr and Ni were determined. The exception is Cd with index values significantly lower on the right side of the noise barrier. The analysis of individual pollution indices for cadmium shows that obtained values exceed values of individual indices for remaining trace metals many times over (Figs. 3 and 4), which were not observed in the analysis of metal concentrations (Fig. 2).

According to Świetlik et al. (2013), road dust collected directly from noise barriers installed on the section of the international road no. E-77 Raszyn-Janki (Poland) reflects the properties of suspended dust from road emissions. The authors estimated that road dust pollution with copper (152 mg/kg; Igeo > 3; PIavg > 20) and zinc (3092 mg/kg; Igeo > 6; PIavg > 140) reached extreme levels, especially in slowed traffic areas. The concentration of nickel was 27.4 mg/kg (Igeo > 1; PIavg < 4), lead at 43.5 mg/kg (Igeo > 1; PIavg < 4), and chromium at 148 mg/kg (Igeo > 2; PIavg < 20). Świetlik et al. (2015) confirmed that the source of zinc in road emissions is the tire and brake wear, diesel exhaust emissions, and corrosion of safety fences. According to the authors, copper comes mainly from abrasion of the brakes, and to a lesser extent, from abrasion of tires and surfaces.

In the study by Różański et al. (2017), the Igeo index was also used in assessing the quality of arable soils along with the distance from the noise screen at the A1 motorway (Grudziądź, Poland). The agricultural land has been classified as moderately polluted from Cd (mean of Igeo of 0.29; maximum of Igeo of 0.56) which results from the 5-year period of road use and the lack of other sources of metal emissions.

**Fig. 5:**
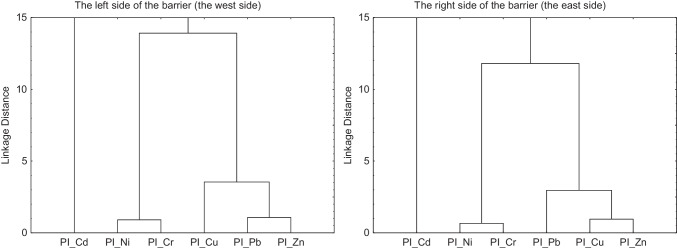
Dendograms of single-pollution index (median values) for trace elements

Identification of trace element clusters in soils along the communication route using the HCA method shows poor aggregation of cadmium with other metals (Fig. 5). The degree of association between the two main groups is at the distance of 272.50 on the left side and 59.36 on the right side of the noise barrier. The clear separation of cadmium from the other five elements is probably associated with high PI values for cadmium (Fig. 4) and very high soil contamination of this element (Table 3). Trace elements with much lower PI values form a separate group, which is further divided into two associated subgroups at the connection spacing of 13.92 (the left side) and 11.80 (the right side). Strong aggregation was noted for copper (3.55) associated with the Pb–Zn group at a bond distance of 1.07 on the left side of the noise screen. On the right side of the barrier, there was a strong aggregation for lead (2.96) linked to the Cu–Zn group at the distance of 0.96. The entire Cu–Zn–Pb subgroup had similar PI values in the range from 3 to 4, except for the maximum of PI points (Table 3, Fig. 4). A high level of aggregation was also observed for the Ni–Cr subgroup (0.90 on the left side and 0.67 on the right side), which indicates the relationship between both trace elements with very low PI values.

Massas et al. (2013) obtained similar conclusions on the relationship of metals in Cu–Zn–Pb and Cr–Ni groups based on the cluster analysis. This indicates the origin of zinc, copper, and lead from road transport, while the cadmium has a different anthropogenic origin in soil (an agricultural activity, emissions from individual heating systems, or the local economic activity related with machining of steel products). In the case of chromium and nickel, values of indices suggest a natural level in soil, and their strong Spearman’s correlations with Zn, Cu, and Pb confirm their common origin, which was also the result of spatial distribution analysis of individual pollution indices (Figs. 3 and 4).

#### Integrated pollution indices

**Fig. 6:**
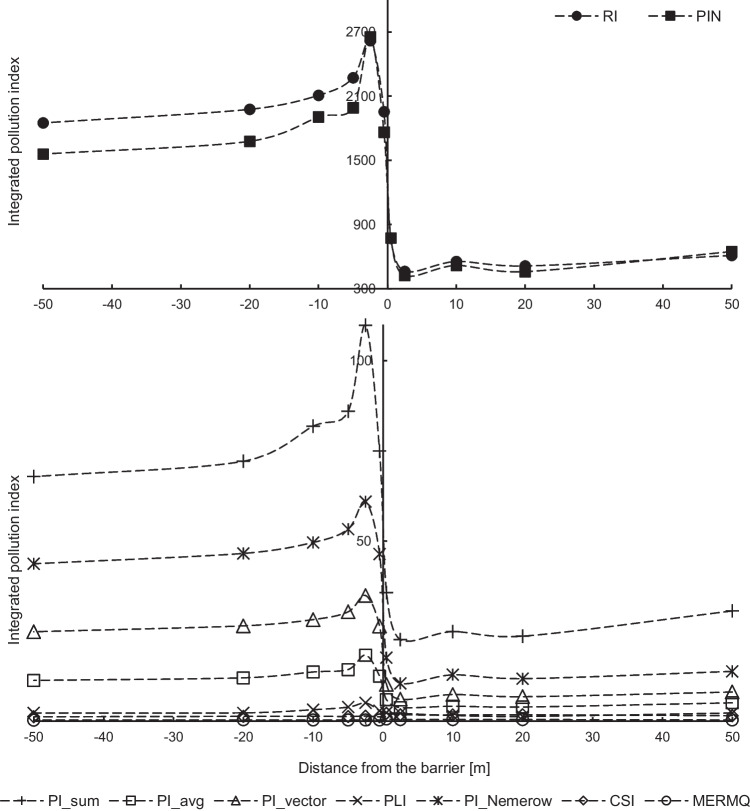
Integrated-pollution indices (median values) of soils with trace elements

Based on PLI and CSI indices, there was only deterioration of soil quality in the studied area due to contamination with trace elements found (Fig. 6). While remaining median values of PIN, PI_average, PI_vector and PI_Nemerow, and RI indices indicate clear, strong pollution of soils and confirm the assessment of individual indices. Based on the MERMQ index, the medium risk level with 21% probability of toxicity was determined for the tested area (Table 4).

**Table 4:** Descriptive statistics of integrated pollution indices

Parameter	PI_sum	PI_avg	PI_vector	PLI	PI_Nemerow	RI	PIN	CSI	MERMQ	Noise barrier	PI_sum	PI_avg	PI_vector	PLI	PI_Nemerow	RI	PIN	CSI	MERMQ
Min	45.42	7.57	14.53	1.21	25.35	1093.48	1000.84	0.41	0.05		9.08	1.51	1.96	0.14	2.63	98.69	81.81	1.17	0.23
Max	273.43	45.57	109.52	8.73	192.39	8070.47	6733.70	4.19	1.34		139.39	23.23	36.86	8.21	62.82	2726.54	3443.34	3.86	1.22
Mean	84.02	14.00	29.66	3.24	52.10	2212.25	1977.94	1.37	0.37		35.34	5.89	10.03	2.32	17.11	743.07	781.59	1.89	0.49
SD	33.96	5.66	12.36	1.60	21.68	915.11	863.20	0.67	0.24		26.88	4.48	7.79	1.57	13.53	585.38	690.16	0.58	0.20
Q1	67.80	11.30	24.48	2.21	43.06	1925.39	1551.77	0.98	0.25		17.72	2.95	4.64	1.24	7.92	343.18	348.96	1.51	0.36
Med	74.95	12.49	27.06	2.76	47.59	2010.73	1720.69	1.30	0.33		28.42	4.74	7.74	2.04	12.88	566.40	595.72	1.74	0.44
Q3	87.40	14.57	30.52	3.88	53.66	2276.01	2042.31	1.57	0.43		40.88	6.81	12.23	2.71	21.11	915.70	913.09	2.04	0.55
Skewnees	3.23	3.23	4.40	1.61	4.42	4.30	3.18	1.84	1.83		2.10	2.10	1.93	1.69	1.86	1.86	2.11	1.75	1.76
Kurtosis	14.37	14.37	26.09	2.61	26.29	25.13	13.71	4.77	4.34		4.76	4.76	3.77	3.45	3.48	3.55	4.74	3.15	3.55
*n*	66	66	66	66	66	66	66	66	66		55	55	55	55	55	55	55	55	55

The spatial distribution of integrated pollution indices (Fig. 6) reflects the trend that is being observed only for cadmium in the distribution of individual pollution indices (Fig. 3 and 4). Therefore, values of integrated pollution indices on the left side of the screen are much higher than on the right side of the screen, both for values calculated based on the PI index and indices calculated from concentrations. This is due to higher by an order of values of PI index for cadmium compared to other elements. Extreme points in Fig. 6 coincide with points in Figs. 2, 3, and 4. In addition, value increases of integrated indices are also observed at 50 m from the screen.

**Fig. 7:**
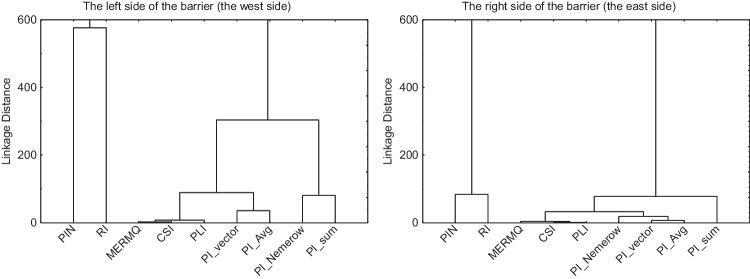
Dendograms of integrated-pollution indices

The cluster analysis suggests which integrated indices should be used to assess the quality of the soil environment. Two main groups of integrated pollution indices are observed in each dendogram (Fig. 7) with a large binding distance between these clusters (~ 15,000 and ~ 4000 of linkage distances, respectively, on the left side and the right side of the barrier). The first group consisted of PIN and RI indices. The second group is further divided into subgroups. On the one side, grouping includes pollution indices that are clearly related to each other, based on the PI index. On the other side, concentration-based indices (CSI, MERMQ) are observed. Therefore, the selection of indices to assess the general level of the soil contamination should include one of selected PI indices (except PI_sum and PI_vector indices due to the lack of soil quality assessment criteria), as well as the MERMQ index due to the clear assessment of a soil quality. The RI index should be calculated independently as it does not have similarities to other integrated pollution indices, and it is the basis for the environmental risk assessment. Calculations of the PIN index based on the contamination classes lead to its separation from the family of PI-based indices (Kowalska et al. 2018).

## Conclusions

The research showed that soil samples collected from both sides of the noise barrier in Wrocław are a good indicator of heavy metal emissions related to road traffic. Noise-absorbing screens affect the transport and spread of pollutants that accumulate in the soil environment. The noise barrier does not completely stop the migration of pollutants adsorbed on the dust, but it has a direct impact on the place of their deposition and concentration in soils. A significant increase in the deposition of trace elements occurs near the barrier, especially on the windward side. Increased metal content was also observed at greater distances from the barrier. Such spatial distribution of metals in studied soils is the result of disturbances caused by the air, which, when passing an obstacle, lifts dust pollution together with trace elements and transports it to greater distances from the barrier. It is recommended to carefully set noise barriers in developed places, especially with densely built-up areas (agglomerations) due to the transport of pollutants at distances above 20 m from the road, which may constitute potential environmental and health risk. Marking bicycle routes along the screens should also consider the direction of winds in the studied area, due to the increased content of air pollutants in the vicinity of the barrier.

The level of metals in studied soils in Wrocław results from the long-term deposition of dust from road emissions. High correlation coefficients between concentrations of trace elements indicate that road emissions are the main source of pollution in the study area. Dendograms for individual indices confirm the common origin of Zn, Cu, and Pb in the soil environment, with high probability from road transport. However, in the case of Cd, there may be an additional anthropogenic source in the study area. The content of Cr and Ni fluctuates at the level of the geochemical background.

The authors in the available scientific studies use only the Igeo index in the assessment of soil contamination at noise barriers. Using individual pollution indices of Igeo and PI in this study, a similar assessment of the quality of soil in Wrocław was obtained. The analysis based on integrated pollution indices shows that it is worth choosing an index from the PI family with clearly defined soil quality assessment criteria and/or an index calculated directly from concentrations (MERMQ). There is no justification for the use of all pollution indices in the assessment of soil contamination, because, for example, PLI and CSI indices assess the degree of soil contamination too mildly and do not confirm the assessment obtained on the basis of other pollution indices, including individual pollution indices. In the case of integrated pollution indices based on the PI index, we receive a direct answer to the degree of soil contamination, which is also confirmed by individual-pollution indices. Although the averaged share of all metals in the integrated index does not clearly indicate the main polluter, in this case, there is Cd and then Zn, Cu, and Pb. To assess the environmental risk, it is useful to calculate the RI index, which includes the toxicity response for a given trace element. Our work shows that pollution indices are a better way of assessing soil quality than relying solely on single-metal concentrations, due to the natural content of metals in the tested soil.

## Data Availability

All data and materials generated or analyzed during this study are included in this article.
